# Supplementary material for: Distinct Oxidative Stress Adaptations Driven by the Overexpression of miR-526b, miR-655, and COX-2 in Breast Cancer
Source: Int J Mol Sci. 2025 Sep 18;26(18):9103. doi: 10.3390/ijms26189103 (PMC12470079; doi:10.3390/ijms26189103)
Supplement: Supplementary file 1 [file ijms-26-09103-s001.zip › ijms-3844225-supplementary.pdf]

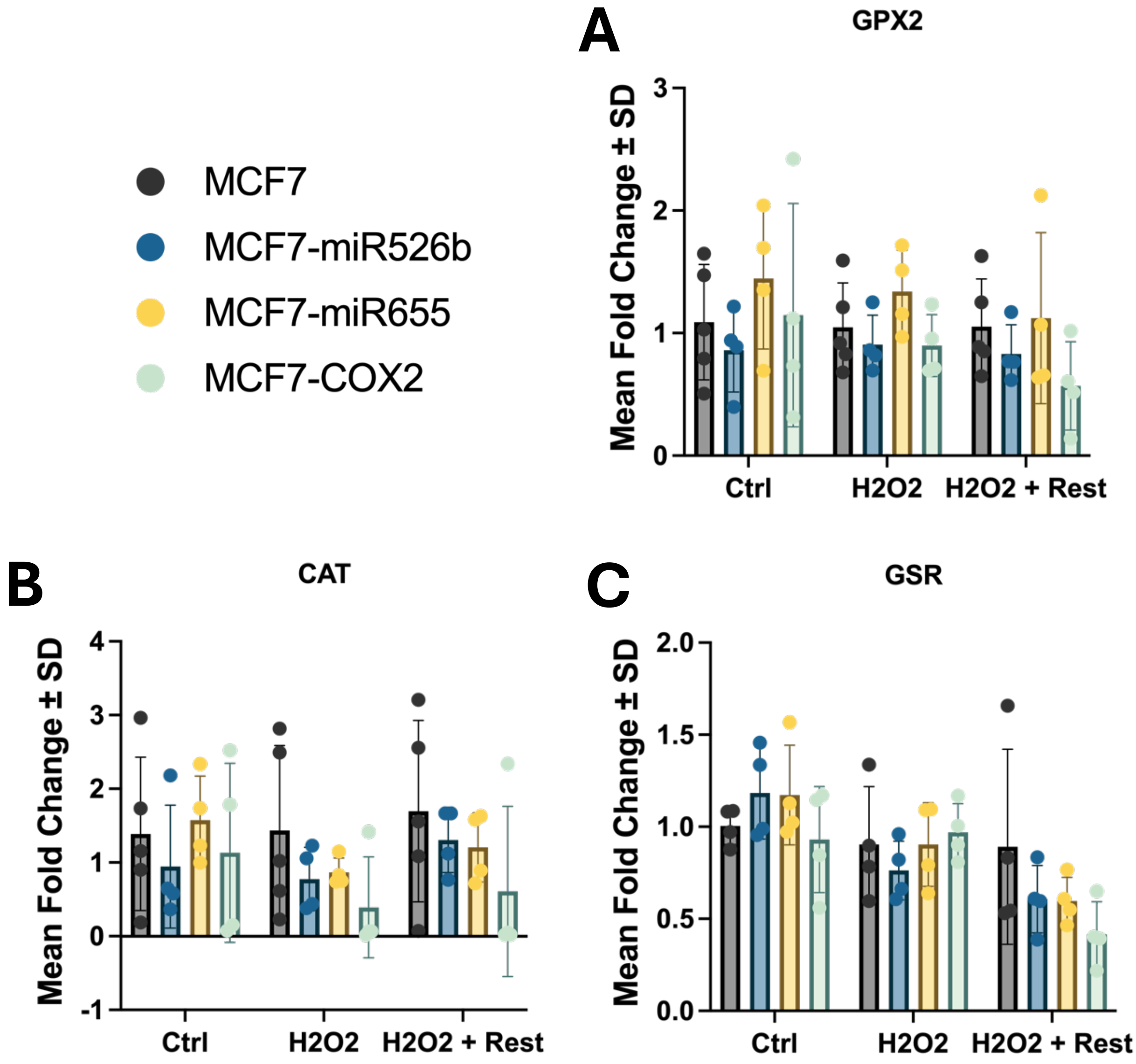

Figure S1: Gene expression of antioxidant markers **A)** GPX2, **B)** CAT and **C)** GSR in H_2O_2-treated cells (MCF7, MCF7-miR526b, MCF7-miR655 and MCF7-COX2).

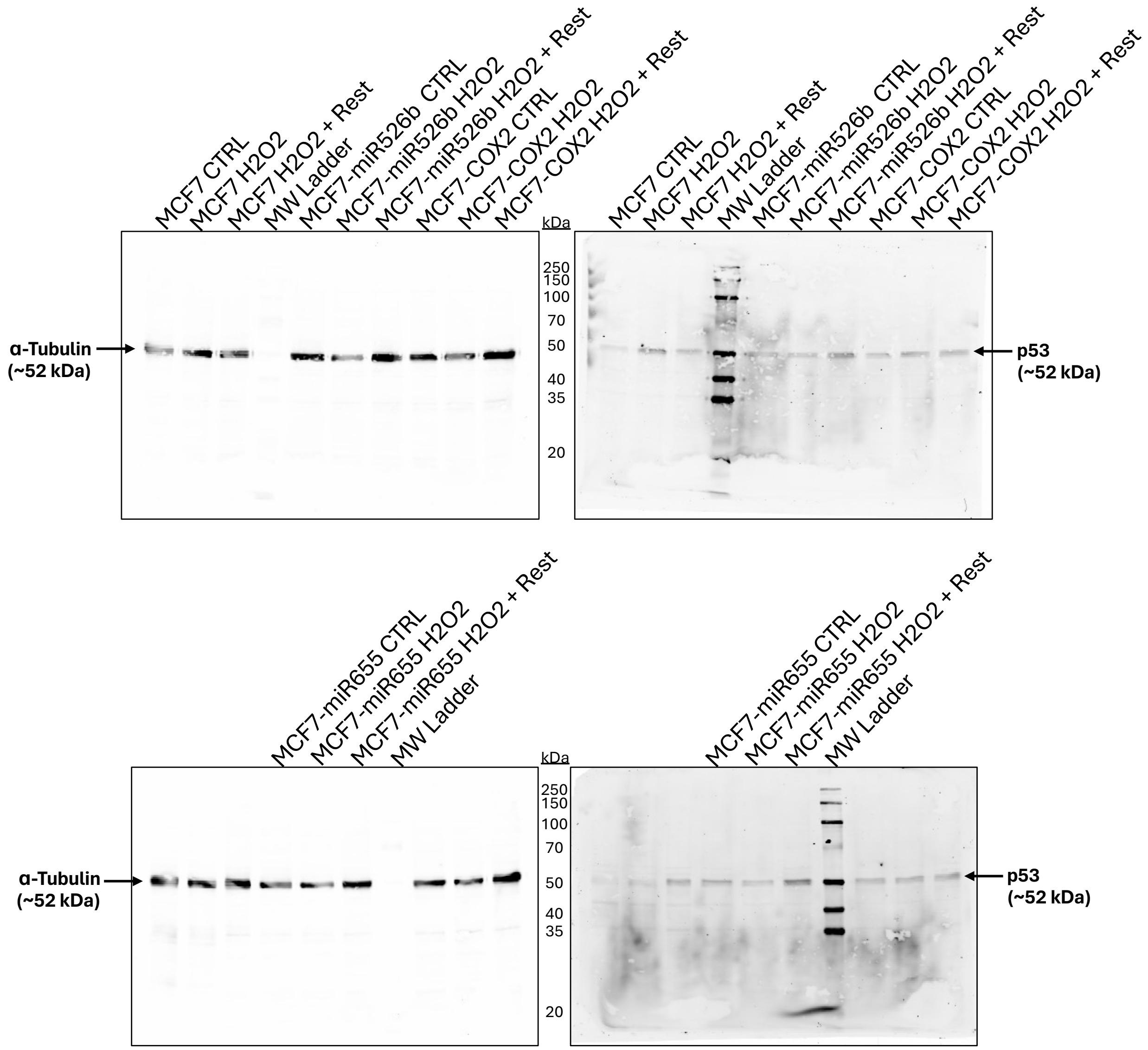

Figure S2: Full western blot images used for representation in figure 1F for p53 and α-Tubulin in H_2O_2-treated cells (MCF7, MCF7-miR526b, MCF7-miR655 and MCF7-COX2). MW: Molecular Weight.

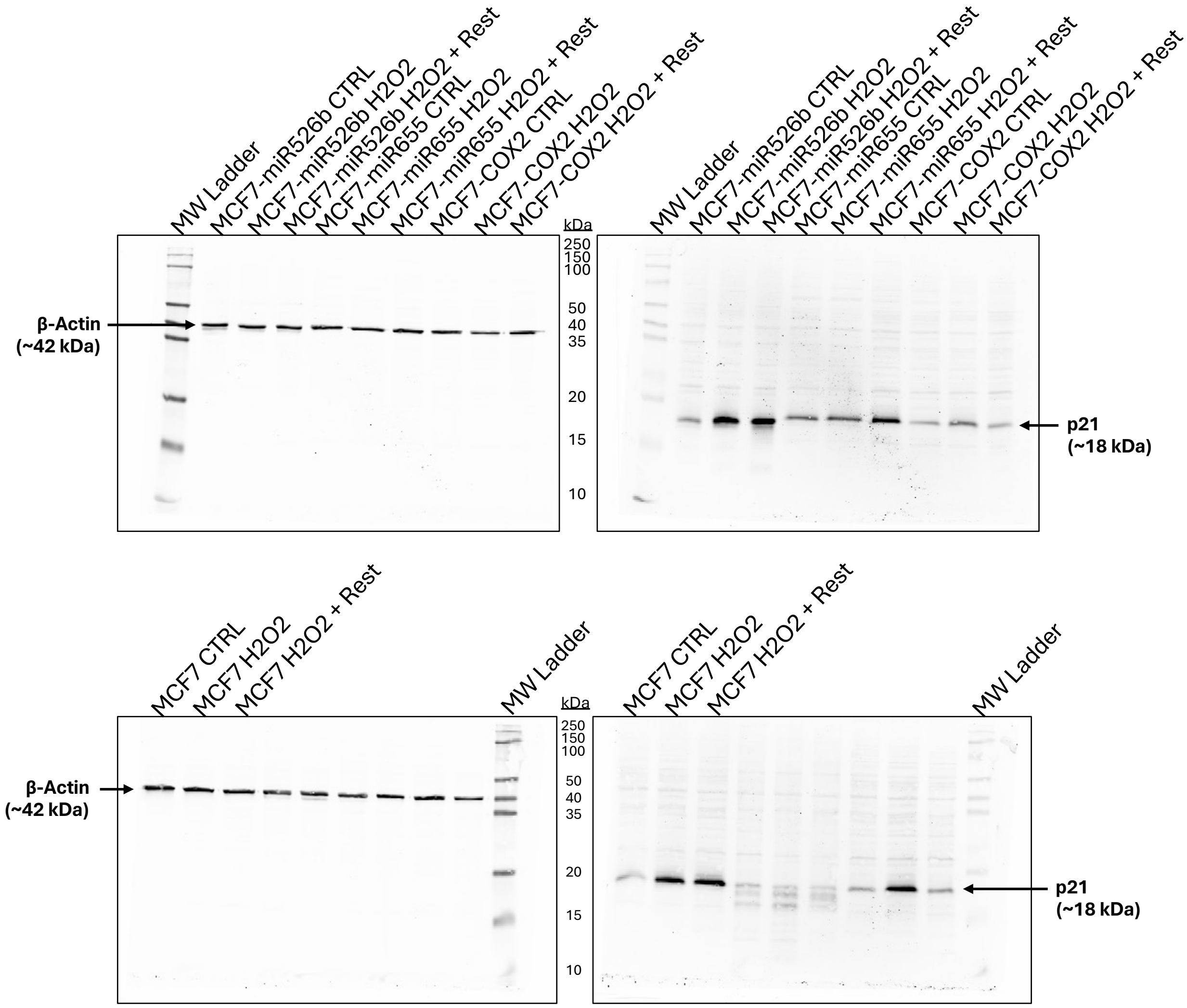

Figure S3: Full western blot images used for representation in figure 1F for p21 and β-Actin in H_2O_2-treated cells (MCF7, MCF7-miR526b, MCF7-miR655 and MCF7-COX2). MW: Molecular Weight.

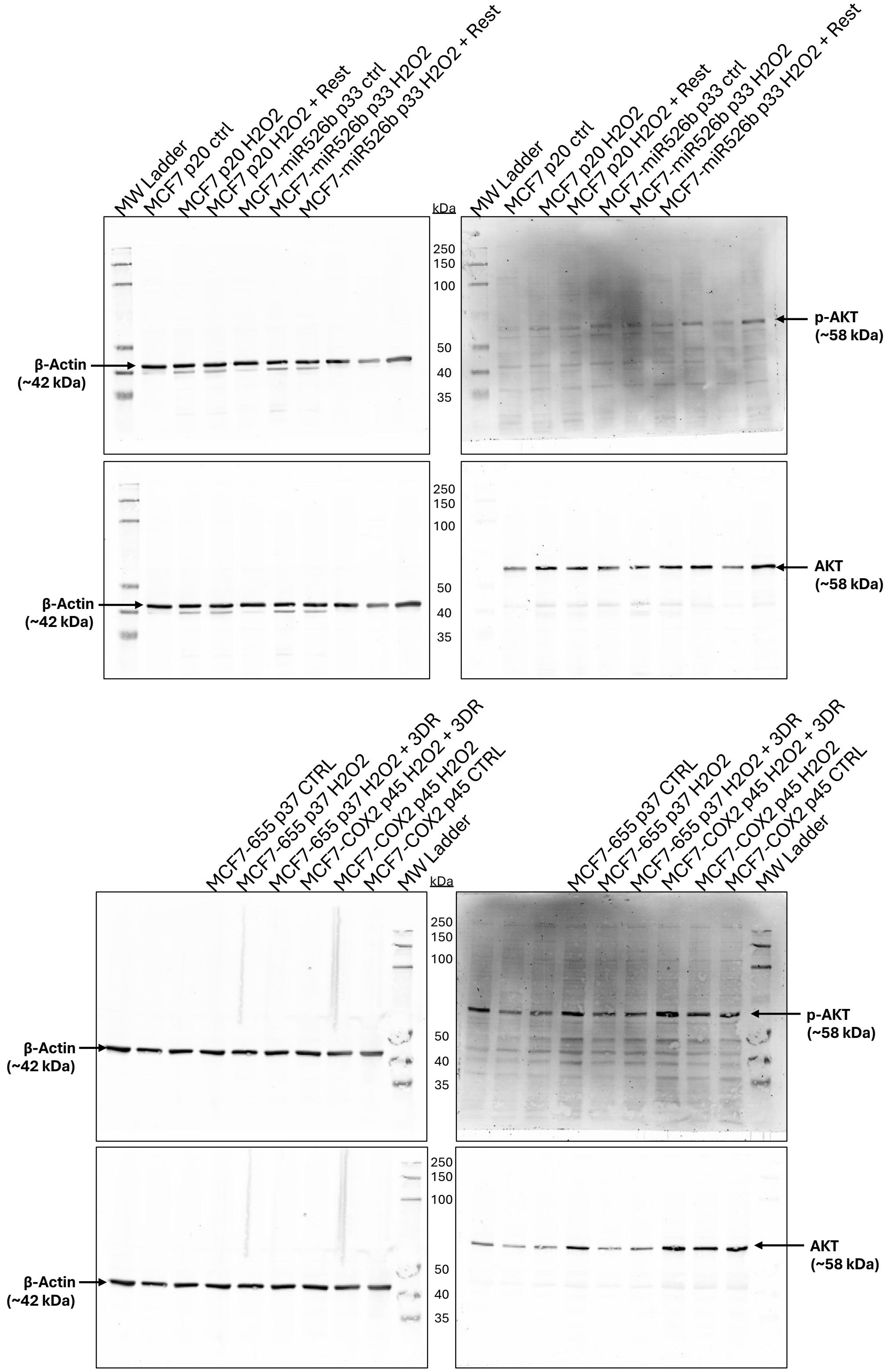

Figure S4: Full western blot images used for representation in figure 1F for total AKT and p-AKT and β-Actin in H_2O_2-treated cells (MCF7, MCF7-miR526b, MCF7-miR655 and MCF7-COX2). MW: Molecular Weight.

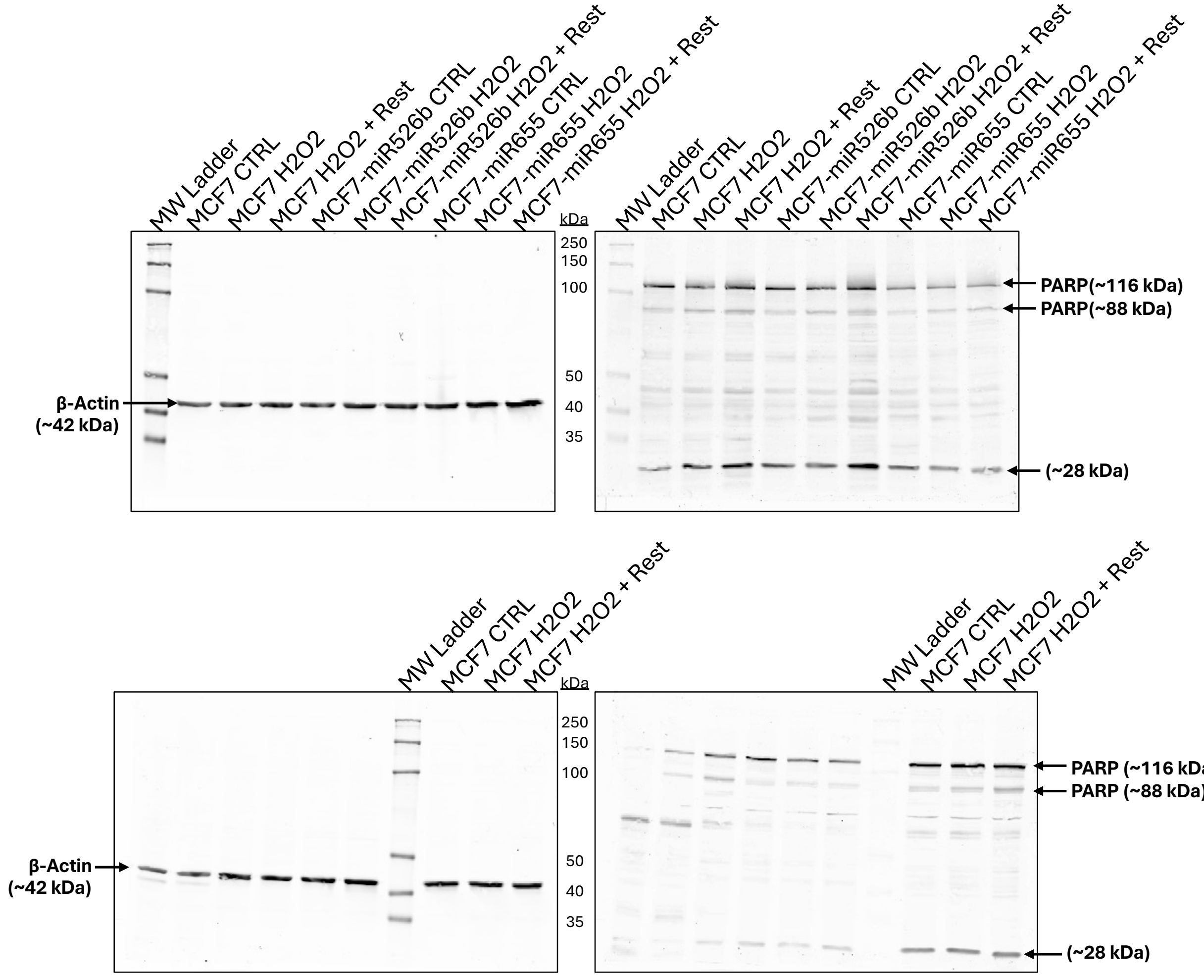

Figure S5: Full western blot images used for representation in figure 1F for full length PARP (116 kDa), fragmented PARP (88 kDa) and β-Actin in H_2O_2-treated cells (MCF7, MCF7-miR526b, MCF7-miR655 and MCF7-COX2). MW: Molecular Weight.

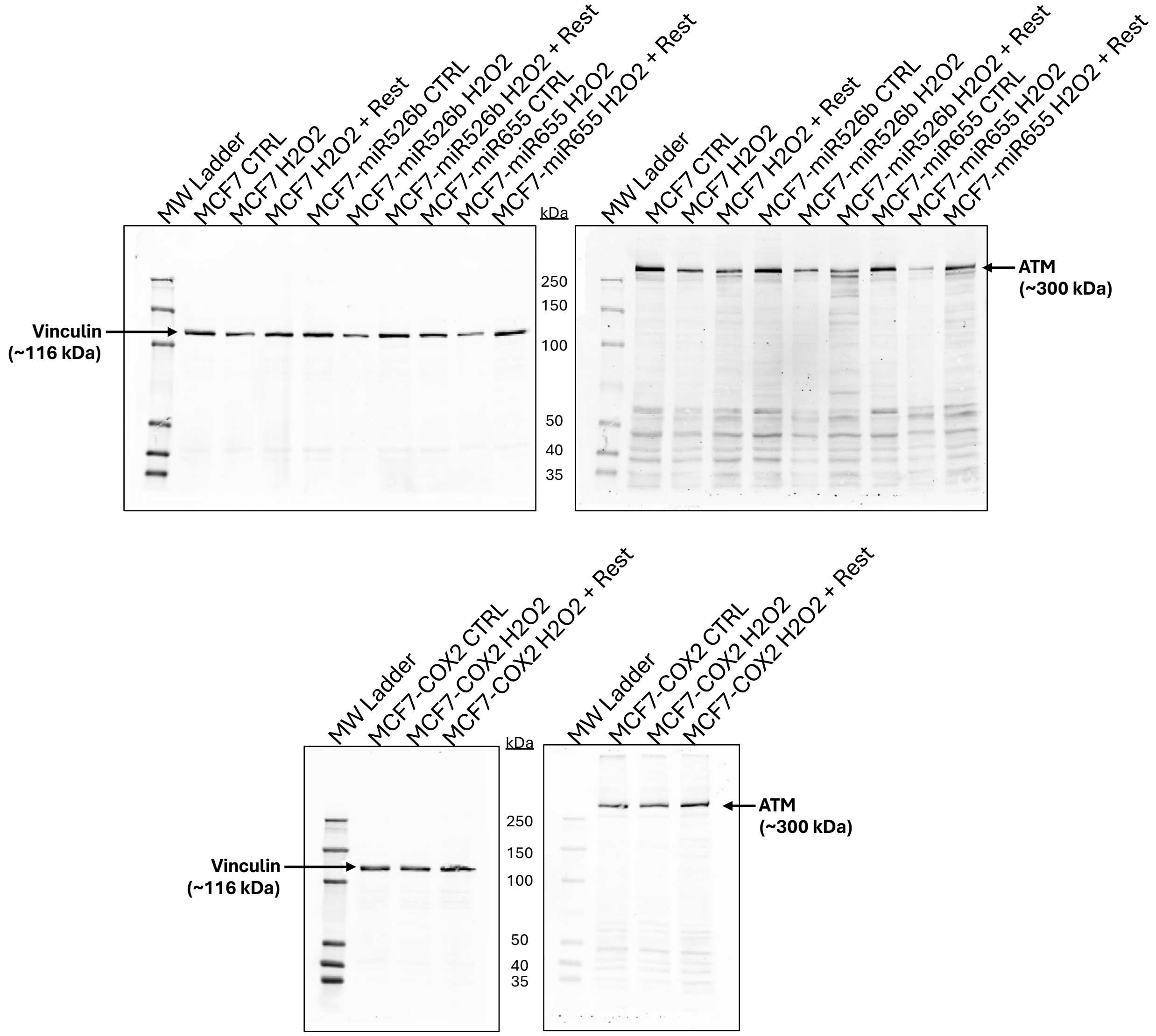

Figure S6: Full western blot images used for representation in figure 3F for ATM and vinculin in H_2O_2-treated cells (MCF7, MCF7-miR526b, MCF7-miR655 and MCF7-COX2). MW: Molecular Weight.

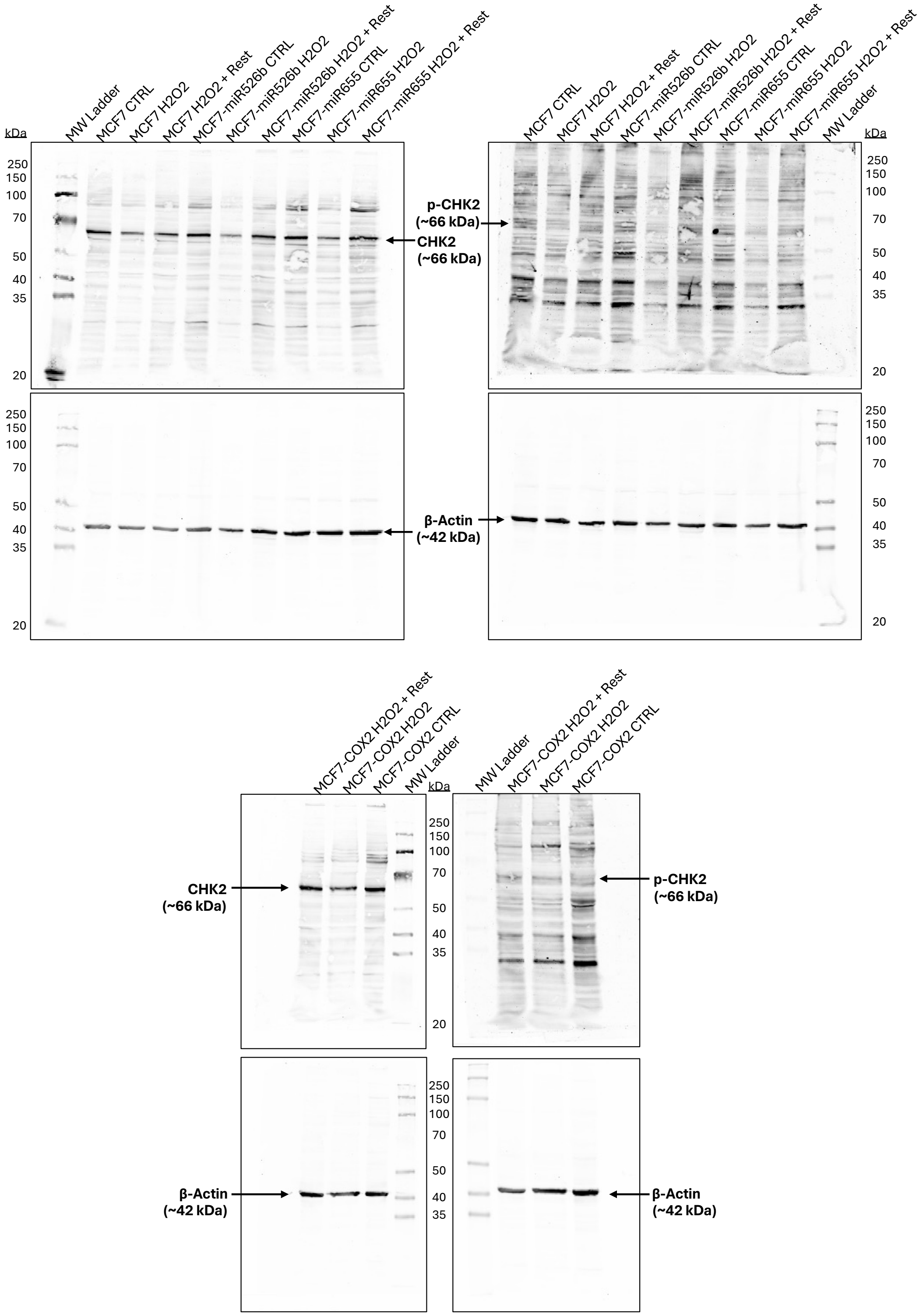

Figure S7: Full western blot images used for representation in figure 3F for CHK2, p-CHK2 and β-Actin in H_2O_2-treated cells (MCF7, MCF7-miR526b, MCF7-miR655 and MCF7-COX2). MW: Molecular Weight.

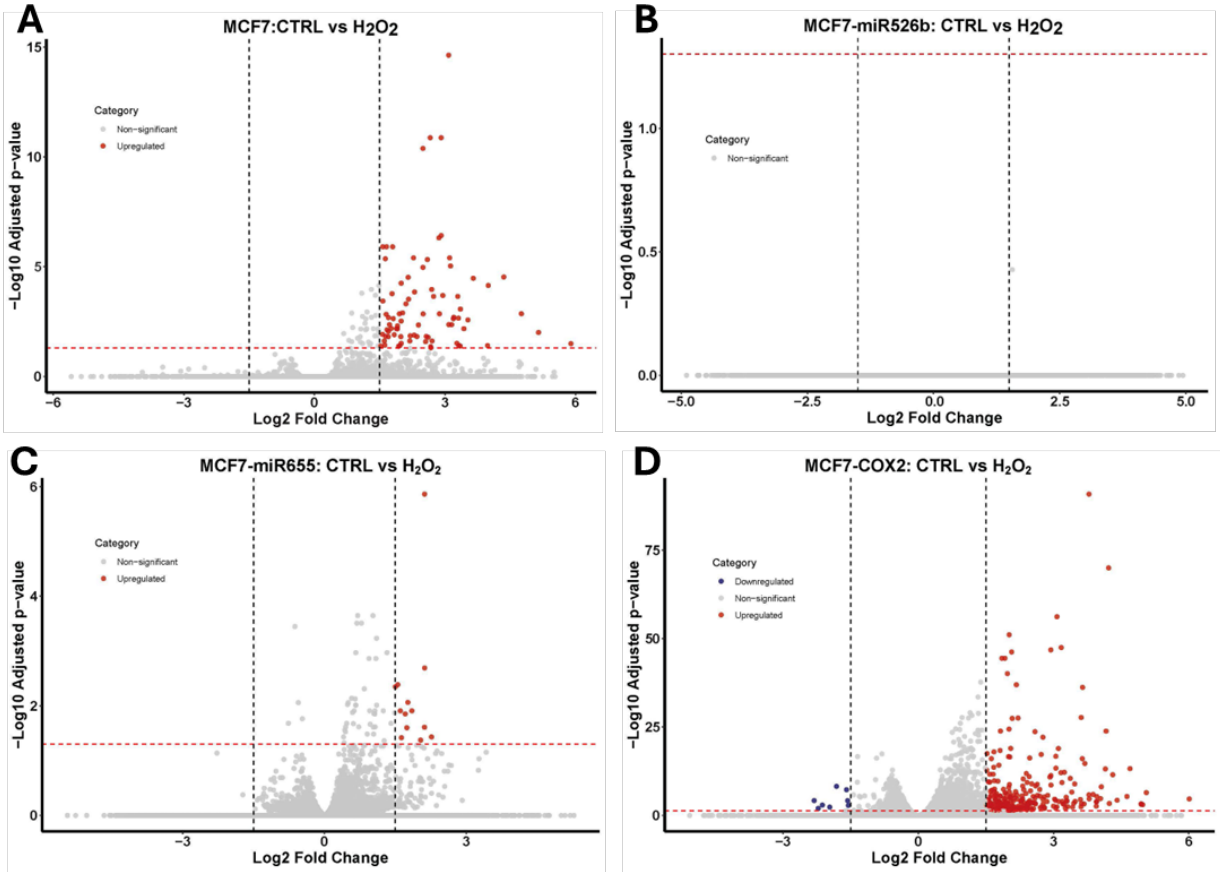

Figure S8: Differential gene expression after H_2O_2 exposure in **A)** MCF7, **B)** MCF7-miR526b, **C)** MCF7-miR655 and **D)** MCF7-COX2. Significance cutoff set to adjusted $p < 0.05$. Log_2 fold change cutoffs set to 1.5 and -1.5.

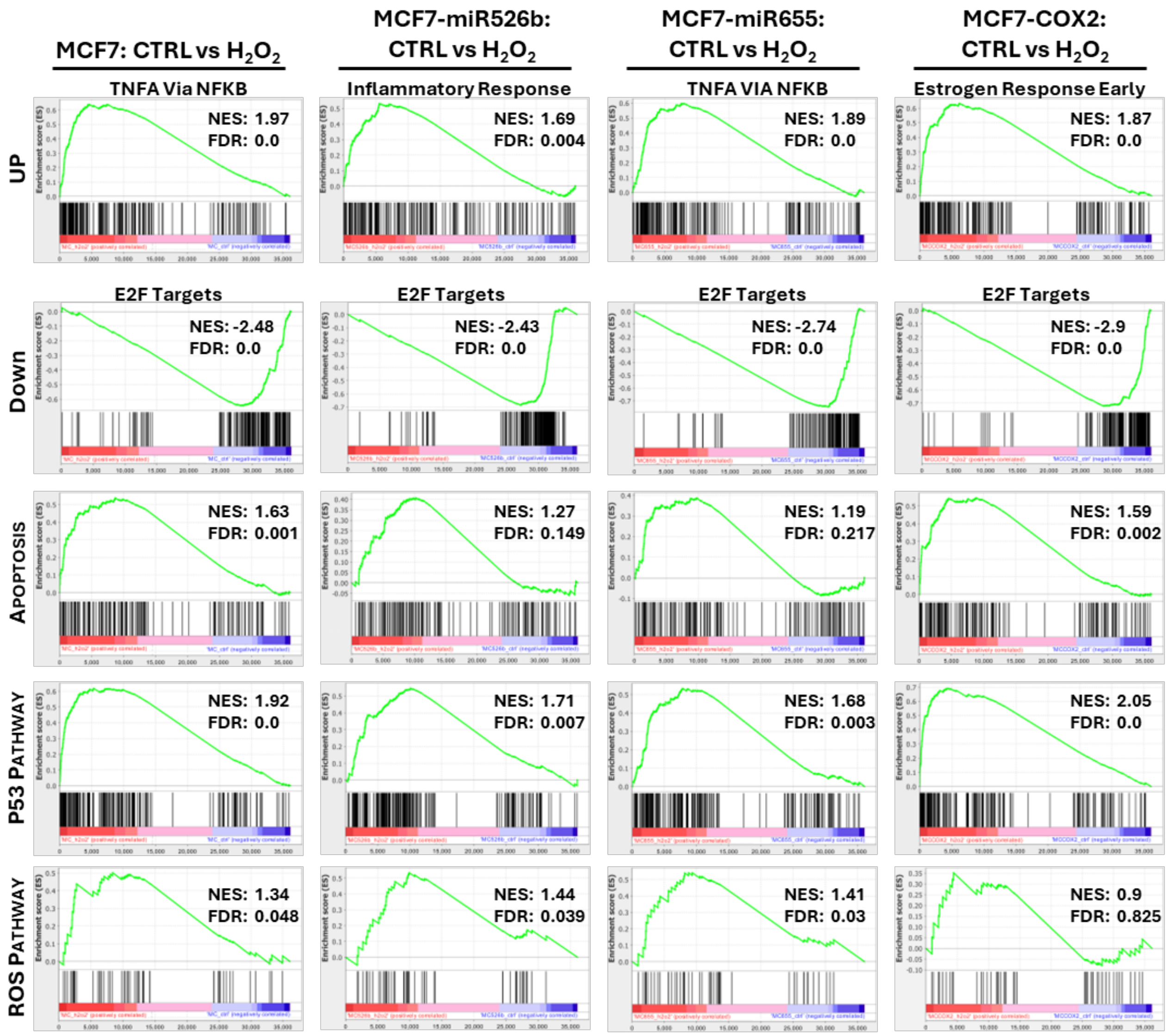

Figure S9: Gene set enrichment analysis after H_2O_2 exposure in MCF7, MCF7-miR526b, MCF7-miR655 and MCF7-COX2. From top to bottom, the greatest positive enriched gene set, the lowest negative enriched gene set, as well as apoptosis, p53 and ROS pathway gene set enrichments.

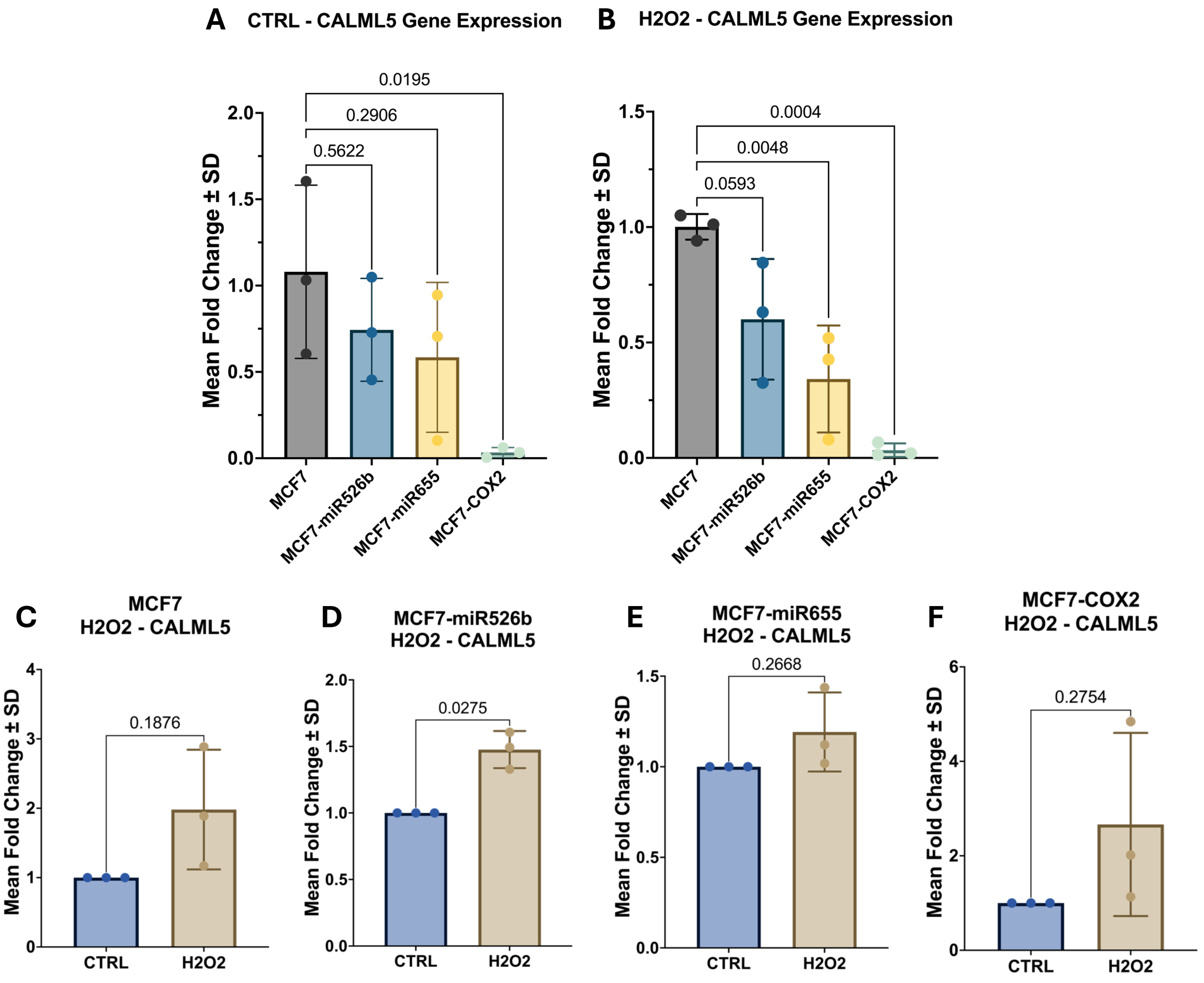

Figure S10: qPCR validation of CALML5 expression. Comparison of CALML5 expression between cell lines in **A)** control (CTRL) and **B)** H_2O_2 conditions (one-way ANOVA). Comparison of CALML5 expression following H_2O_2 exposure in **C)** MCF7, **D)** MCF7-miR526b, **E)** MCF7-miR655 and **F)** MCF7-COX2 (paired t-test).
